# Pro198Leu Polymorphism in the Glutathione Peroxidase 1 Gene Contributes to Diabetic Peripheral Neuropathy in Type 2 Diabetes Patients

**DOI:** 10.1007/s12017-016-8438-2

**Published:** 2016-09-03

**Authors:** Monika Buraczynska, Kinga Buraczynska, Michal Dragan, Andrzej Ksiazek

**Affiliations:** 1 Department of Nephrology, Medical University of Lublin, Dr K. Jaczewskiego 8, 20-954 Lublin, Poland; 2 Department of Neurology, Medical University of Lublin, Lublin, Poland

**Keywords:** Gpx1, Type 2 diabetes, Peripheral neuropathy, Pro198Leu polymorphism, Risk allele

## Abstract

Glutathione peroxidase 1 (Gpx1) is an endogenous antioxidant enzyme. The T allele of the Pro198Leu polymorphism in the Gpx1 (rs1050450, 198C > T) gene is associated with reduced enzyme activity. The aim of this study was to evaluate the association between Pro198Leu polymorphism and risk of diabetic peripheral neuropathy (DPN). We examined 1244 T2DM patients and 730 healthy controls. In the patient group, 33 % had diabetic peripheral neuropathy. All subjects were genotyped for the Gpx1 Pro198Leu polymorphism by polymerase chain reaction and restriction analysis. A significant increase in the T allele and TT genotype frequencies was observed in DPN patients compared to those without DPN (OR 1.55, 95 % CI 1.30–1.85 and 1.89, 95 % CI 1.30–2.74, respectively). The association remained significant after correction for age, disease duration, HbA1c and BMI. When distribution of T allele was compared between DPN+ and DPN− subgroups and controls, OR was 1.54 for DPN+ and 1.00 for DPN− patients. In conclusion, our findings suggest that Gpx1 Pro198Leu genotypes are significantly associated with the risk of diabetic peripheral neuropathy in patients with T2DM. The study provides new clinically relevant information regarding genetic determinants of susceptibility to diabetic neuropathy.

## Introduction

Diabetes mellitus (DM) is a chronic multifactorial disease characterized by persistent hyperglycemia that is associated with increased morbidity and mortality. Increased mortality in diabetes is due to the development of both macrovascular and microvascular complications (Brownlee 2005). One of the frequent late microvascular complications of diabetes is diabetic neuropathy (Vinik et al. 2000; Vincent et al. 2004). At least 50 % of patients with type 2 diabetes suffer from the most common form, diabetic peripheral neuropathy (DPN) (Kasznicki et al. 2012). It is characterized by inflammation and degeneration of peripheral nerves and accompanied by pain, loss of sensation and paresthesia. It predisposes to severe functional limitations and serious complications including leg amputation (Ybarra et al. 2010; El Bghdady and Badr 2012).

DPN has a complex pathogenesis, involving vascular, metabolic and neurochemical processes. Chronic hyperglycemia is involved in the development of mitochondrial dysfunction, leading to reactive oxygen species (ROS) overproduction (Brownlee 2001). Additional sources of ROS in diabetes include glucose autooxidation, protein kinase C activation, methylglyoxal formation and glycation, hexosamine metabolism and sorbitol formation. All these factors contribute to excessive oxidative stress (Evans et al. 2002; Robertson et al. 2004). In type 2 diabetes, the activity of major antioxidant enzymes such as superoxide dismutase (SOD), glutathione peroxidase (GPx) and catalase (CAT) is altered (Faure et al. 2008). The single-nucleotide polymorphisms in genes coding for antioxidant enzymes may be associated with decreased or impaired ROS detoxification and increase oxidative damage (Flekac et al. 2008). Molecular variants of antioxidant enzymes are potential risk factors for chronic complications of diabetes (Forsberg et al. 2001; Houldsworth et al. 2015).

Human glutathione peroxidase (GPX) is an antioxidant enzyme expressed in blood vessels. Its main role is protecting cells against oxidative damage by reducing hydrogen peroxide and organic peroxidases to H_2_O_2_ with reduced glutathione (Suzen et al. 2010). The most common isoform of GPS family is GPX1, a selenium-dependent enzyme encoded by Gpx1 gene. The gene is located on chromosome 3p21 and contains two exons. One of its polymorphisms is a leucine to proline change at codon position 198 (Jefferies et al. 2005). The Leu allele was reported to be associated with genetic susceptibility to coronary atherosclerosis in type 2 diabetic patients (Nemoto et al. 2007).

The purpose of the present study was to evaluate the association of the Pro197Leu polymorphism in the Gpx1 gene with diabetic neuropathy in type 2 diabetes patients. Our hypothesis was that this polymorphism in the gene encoding antioxidant enzyme could be associated with diabetes and/or its complications.

## Materials and Methods

### Subjects

The study population included 1244 unrelated T2DM patients, consecutively enrolled between 2008 and 2014 from the Departments of Nephrology and Cardiology of Medical University of Lublin. All subjects were Caucasians of Polish origin. The type 2 diabetes was diagnosed according to American Diabetes Association criteria (American Diabetes Association 2013). Diagnosis was based on one or more of the following conditions: classic symptoms of hyperglycemia (polyuria, polydipsia, weight loss), random plasma glucose ≥ 11 mmol/L or fasting plasma glucose ≥ 7 mmol/L or and/or treatment with antidiabetic agents. Age at diagnosis of T2DM was > 35 years in all subjects. The mean duration of diabetes was 12.8 years (range 7–31). Cardiovascular disease was diagnosed in 888 patients (71 %) as one or combination of pathological states: ischemic heart disease (chronic coronary heart disease and/or acute coronary syndromes), congestive heart failure or ischemic cerebral stroke. Clinical manifestations of CVD were confirmed by relevant biochemical, radiographic, echocardiographic and vascular diagnostic criteria. Among the patients 946 individuals (76 %) were hypertensive, according to World Health Organization criteria. Diabetic nephropathy was diagnosed clinically in the presence of persistent albuminuria ≥ 300 mg/24 h in at least two consecutive determinations in the absence of hematuria or infection. Diabetic retinopathy was diagnosed according to the Early Treatment Diabetic Retinopathy Study (ETDRS) criteria: the presence of microaneurysms, hemorrhages, cotton wool spots, intraretinal microvascular abnormalities, hard exudates, venous beading and new vessels. In T2DM patient group, 406 individuals (32.6 %) had diabetic neuropathy. Neurological assessment included evaluation of neuropathic symptoms and deficits. Diabetic neuropathy was diagnosed by clinical neurological examination and electromyography (Viking Select EMG System, Nicolet Biomedical, USA) according to the recent guidelines (Tesfaye et al. 2010).

Healthy control subjects (*n* = 730) with normal ECG and no clinical signs of renal, cardiovascular and neurological disease were randomly recruited among hospital staff and blood bank donors who underwent health examination. They were asked about risk factors, existing diseases and medication intake. Subjects with positive family history of DM or CVD in first degree relatives were excluded. Before the inclusion into the study, a written informed consent for genotyping was obtained from all patients and controls in accordance with principles of the 1964 Declaration of Helsinki. The institutional ethics committee approved the protocol of the study.

### Determination of Gpx1 Pro198Leu Genotype

Genomic DNA was extracted from peripheral blood leukocytes obtained by the standard procedure. The Pro198Leu polymorphism in the Gpx1 gene (rs1050450, 198C > T) was analyzed by amplification of 337-bp DNA fragment by polymerase chain reaction (PCR). The following primers were used for amplification reaction: sense primer 5′-TGTGCCCCTACGGTACA-3′ and antisense primer 5′-CCAAATGACAATGACACAGG-3′ (Hu and Diamond 2003). Genomic DNA (300 ng) was amplified in a final volume of 30 μl using the following conditions: initial denaturation at 95 °C for 5 min, followed by 35 cycles of 94 °C for 30 s, annealing at 58 °C for 30 s and extension at 72 °C for 2 min. A final extension step was at 72 °C for 10 min. The PCR product containing polymorphic site was 338-bp fragment. Ten μl of the PCR product were digested overnight at 37 °C with 5 U of Apa I restriction endonuclease (Thermo Fisher Scientific, Waltham, MA), and resulting fragments were separated on a 2 % agarose gel. The fragment sizes were 259 + 79 bp for wild type (CC), 338 + 258 + 79 bp for CT heterozygote and 338-bp undigested fragment for polymorphic variant (TT). The quality of genotyping was controlled by using blind DNA duplicates for random samples. In addition, 20 samples were randomly selected for each genotype, and the PCR products were sequenced in CEQ 8000 Genetic Analysis System (Beckman Coulter, England). There was a 100 % concordance between genotyping assays.

### Statistical Analysis

Statistical calculations were performed using Statistical Package for Social Sciences version 11.0 for Windows (SPSS, Inc., Chicago, IL, USA). For baseline characteristics, the normally distributed continuous variables are presented as mean ± SD. The Hardy–Weinberg equilibrium was assessed using a Χ^2^ test with 1 degree of freedom. Genotype distribution and allele frequencies were compared between groups using a Pearson χ^2^ test of independence with 2 × 2 contingency and *z* statistics. Analysis of variance (ANOVA) was used to compare the distribution of quantitative biochemical parameters. Pearson *Χ* ^2^ test and Mann–Whitney test were used for comparing discrete and continuous variables. For significant allelic and genotyping associations, the adjusted odds ratios (ORs) with corresponding 95 % confidence intervals (CIs) were calculated. In the T2DM DPN+ patient group, the frequency of the Pro198Leu T allele was 0.37. The study had 97.2 % power (α = 0.05) to detect an association (OR vs DPN− 1.55, 95 % CI 1.30–1.85). An interaction of the polymorphism with various risk factors was examined with unconditional model of multiple logistic regression analysis. All tests were two-sided with statistical significance set at *p* < 0.05.

## Results

In this case–control study, the Pro198Leu polymorphism (rs1050450) in the Gpx1 gene was genotyped in 1244 patients with T2DM (33 % had diabetic neuropathy) and 730 healthy control subjects. Baseline demographic and clinical characteristics of T2DM patients are summarized in Table 1. Age at study and gender did not statistically differ between patients with diabetic neuropathy and those without it. HbA1c was higher in DPN subgroup when compared with patients without DPN (*p* = 0.028). As expected, the diabetes duration was longer in the DPN subgroup than in patients without DPN (*p* < 0.0001). The DPN subgroup had a higher prevalence of other microvascular complications (*p* < 0.0001). The patients with DPN were about 4 years younger at diabetes onset than those without DPN (*p* < 0.0001). The prevalence of cardiovascular disease differed significantly between the two subgroups (*p* = 0.027).

**Table 1:** Demographic and clinical profile of studied subjects

Variable	T2DM with DPN	T2DM without DPN	*P* value^a^
	(*n* = 406)	(*n* = 838)	
Male (%)	194 (47.8)	400 (47.7)	0.973
Age at study (years)	63 ± 12.4	62.9 ± 13.5	0.900
Age at diabetes onset (years)	48.3 ± 15.8	52 ± 14.3	<0.0001
Diabetes duration (years)	14.9 ± 9.7	10.7 ± 8.7	<0.0001
Cardiovascular disease (%)	310 (76.3)	578 (69.3)	0.027
Essential hypertension (%)	322 (80)	624 (75.7)	0.155
Other MV complications (%)	320 (78.8)	378 (45.1)	<0.0001
Family history of diabetes (%)	174 (42.9)	341 (47)	0.460
HbA1c (%)	8.3 ± 1.9	8.1 ± 1.5	0.028
Total cholesterol (mmol/l)	4.6 ± 1.7	4.4 ± 1.9	0.072
HDL cholesterol (mmol/l)	1.2 ± 0.45	1.2 ± 0.38	1.000
LDL cholesterol (mmol/l)	2.8 ± 1.05	2.8 ± 1.09	1.000
Triglycerides (mmol/l)	1.8 ± 1.08	1.8 ± 1.15	1.000
BMI (kg/m^2^)	29.6 ± 6.5	29.1 ± 5.2	0.144

*T2DM* type 2 diabetes mellitus, *DPN* diabetic peripheral neuropathy, *MV* microvascular

Values are presented as mean ± SD (continuous characteristics) or as numbers with percent in parentheses (discrete characteristics)

^a^ Pearson’s χ^2^ test for categorical variables, Mann–Whitney test for continuous variables

The prevalence of the Pro198Leu (C/T) genotype and allele frequencies in type 2 diabetes patients and controls is presented in Table 2. The observed frequencies of the T allele and TT genotype were different between these groups (OR 1.16, *p* = 0.041 and OR 1.40, *p* = 0.034, respectively). We next compared distribution of alleles and genotypes between patients with DPN and patients without it. A significant increase in the T allele frequency was observed in DPN patients compared to those without DPN (37vs 27 %, *p* = 0.0003) (Table 3). The variant T allele was associated with a higher risk of developing DPN (OR 1.55, *p* < 0.0001). Both genotypes, the homozygous TT and heterozygous CT, had even greater effect (OR 1.89, *p* = 0.0008 and OR 1.78, *p* < 0.0001, respectively). The association remained significant after correction for age, disease duration, HbA1c and BMI. When distribution of the T allele was compared between DPN+ and DPN− subgroups of patients and controls, for DPN+ OR was 1.54, *p* < 0.0001 and for DPN− OR 1.00, *p* = 0.9947.

**Table 2:** Genotype and allele distribution of Pro198Leu polymorphism in Gpx1 gene in T2DM patients and controls

		Genotypes			Alleles		OR (95 % CI)	
	N	CC	CT	TT	C	T	TT genotype^a^	T allele
T2DM patients	1244	635 (51)	460 (37)	149 (12)	0.70	0.30	1.40 (1.02–1.93)	1.16 (1.00–1.34)
							*p* = 0.034	*p* = 0.041
Controls	730	396 (54)	268 (37)	66 (9)	0.73	0.27	Ref.	Ref.

*T2DM* type 2 diabetes mellitus

Genotype distributions are shown as numbers (%)

^a^ Calculated versus CC genotype

**Table 3:** Genotype and allele distribution of Pro198Leu polymorphism in Gpx1 gene in T2DM patients with and without DPN

		Genotypes			Alleles		OR (95 % CI)		
	N	CC	CT	TT	C	T	TT genotype^a^	CT genotype^a^	T allele
T2DM with DPN	406	167 (41)	179 (44)	60 (15)	0.63	0.37	1.89 (1.30–2.74)	1.78 (1.38–2.30)	1.55 (1.30–1.85)
							*p* = 0.0008	*p* < 0.0001	*p* < 0.0001
T2DM without DPN	838	468 (56)	281 (33)	89 (11)	0.73	0.27	Ref.	Ref.	Ref.

Genotype distribution is shown as numbers (%)

*T2DM* type 2 diabetes, *DPN* diabetic peripheral neuropathy

^a^ OR calculated versus CC genotype. The ORs adjusted for age, disease duration, HbA1c and BMI: for TT genotype OR 1.56 (1.04–2.63), *p* = 0.011; for CT genotype OR 1.49 (0.71–2.4), *p* = 0.008; for T allele 1.37 (0.66–2.51), *p* = 0.001. The study had 97.2 % power (α = 0.05) to detect an association (OR versus DPN− 1.55, 95 % CI 1.30–1.85)

In logistic regression analysis regarding the presence of DPN and different variables: age, gender, duration of diabetes, hypertension, total cholesterol, HDL cholesterol, triglycerides and BMI no statistical associations were observed (Table 4).

**Table 4:** Multiple logistic regression analysis

Variable	OR (95 % CI)	*P*
Age	1.18 (0.83–1.39)	0.078
Gender	1.12 (0.91–1.22)	0.137
Age at diabetes onset	1.09 (0.91–1.32)	0.091
Disease duration	0.98 (0.89–1.06)	0.082
Cardiovascular disease	1.27 (0.85–1.92)	0.144
Hyperlipidemia	1.36 (0.69–2.82)	0.262
HbA1c	1.15 (0.99–1.41)	0.084
BMI	1.02 (0.96–1.09)	0.832
TT genotype^a^	2.13 (1.42–4.03)	0.009

An unconditional model of multiple logistic regression analysis was used for interaction of the polymorphism with various risk factors

^a^ Pro198Leu polymorphism

Since the Pro198Leu polymorphism in the Gpx1 gene was previously reported to be associated with cardiovascular disease in diabetic subjects, we checked the distribution of this polymorphism in subgroups of CVD + and CVD − patients. No statistically significant differences were observed between the subgroups. In CVD + patients versus CVD − the OR for T allele was 0.97 (0.80–1.17), *p* = 0.767 and for TT genotype 0.99 (0.66–1.47), *p* = 0.969.

## Discussion

Diabetic peripheral neuropathy is common complication of diabetes with complex, multifactorial pathogenetic mechanisms (Vinik et al. 2013). Analysis of candidate gene variants in DPN could be useful in identifying patients susceptible to development of this complication. Oxidative stress is involved in the development of diabetes and its complications (Robertson et al. 2004; Vincent et al. 2004). Several studies have shown that the Gpx1 Pro198Leu polymorphism increases the incidence of oxidative stress-related diseases (Tang et al. 2008; Matsuno et al. 2011; Chen et al. 2012; Ramprasath et al. 2012; Cao et al. 2014).

In the present study, we identified an association between the Pro197Leu polymorphism in the Gpx1 gene and diabetic peripheral neuropathy in type 2 diabetes patients. The main finding is that the T allele of the Pro198Leu polymorphism might increase risk of DPN. The similar result was obtained by Tang et al. (2012). In a study of two independent samples of Caucasian patients with diabetes mellitus, the authors observed a significant association between the T allele of the Pro198Leu polymorphism and peripheral neuropathy. This association was independent of other risk factors. In a small Japanese study of 173 type 2 diabetes patients, the Pro198Leu polymorphism in Gpx1 gene was found to be a predisposing factor in distal symmetric polyneuropathy and macrovascular disease (Matsuno et al. 2011). Our results are not consistent with a previous study performed in a Polish population that showed no association between the Gpx1 Pro198Leu polymorphism and distal symmetric polyneuropathy in patients with type 2 diabetes (Kasznicki et al. 2016). It is difficult to explain this discrepancy between studies. Possibly part of it might be a number of patients studied. Our study involved 1244 T2DM patients versus 245 in Kasznicki et al. study. Diabetic neuropathy is a complex condition in which it might be difficult to demonstrate an impact of a single polymorphism. An interactive effect of several factors may lead to underestimation or overestimation of a role of given polymorphism in determining the phenotype. What’s more, control subjects are considered free of diabetic neuropathy by medical history, lack of neurological deficits and laboratory examinations. This does not exclude subjects who might be affected by subclinical neuropathy.

In our study, there was no significant association with the polymorphic variant of the Pro198Leu with cardiovascular disease. This is in line with other reports from Caucasian populations (Tang et al. 2012; Kasznicki et al. 2016). Such association was reported in the studies from Asia (Hamanishi et al. 2004; Tang et al. 2008). This discrepancy may be due to the ethnic and baseline characteristics differences between populations.

Some studies reported an association with Pro198Leu polymorphism in Gpx1 with type 2 diabetes (Ramprasath et al. 2012). We also observed an association with the Pro198Leu T allele with diabetes itself when compared to healthy control subjects. In a Japanese study, the Pro198Leu polymorphism was associated with the development of diabetic polyneuropathy but not the onset of diabetes itself (Matsuno et al. 2011). A similar result was observed in the study of Vats et al. (2015).

The mechanism of the observed effect of Gpx1 gene polymorphism on susceptibility to diabetic neuropathy is unknown. Several studies confirmed the association with Pro198Leu with Gpx1 activity (Ravn-Haren et al. 2006; Hansen et al. 2009). Changing the capacity of antioxidant enzyme may lead to increased oxidative damage associated with the T allele. Previous studies have shown a link between Gpx activity and LDL oxidation (Rosenblat and Aviram^1998^). This was found to be important in the etiology of microvasculitis and local ischemic injury of peripheral nerves (Said et al. 2003). Also, increased OxLDL/apoB ratio was associated with peripheral neuropathy in type 2 diabetes (Tsuzura et al. 2004). One of the possible pathophysiological mechanisms involved in Gpx1 role in peripheral neuropathy might be an impaired microcirculation in the peripheral nerves caused by vascular endothelial dysfunction associated with oxidative stress (Hamanishi et al. 2004). A direct nerve damage resulting from elevated oxidative stress is also possible (Oyenihi et al. 2015).

Our study has some limitations. It is a retrospective case–control study; thus, a selection bias cannot be excluded. To limit this bias, we included consecutive patients in the study and tried to adjust for known confounding risk factors. We were unable to score the severity of neuropathy in the entire patient cohort and therefore to perform subgroup analyses in patients with different severity scores. We also did not examine the association between Gpx1 genotype and enzyme activity in our patient cohort and we plan to analyze this in our further work. Also, we examined only one polymorphism in the Gpx1 gene. The strength of our study is that all patients and controls are of the same ethnic origin. Furthermore, all subjects were examined in a standardized manner, with well defined diagnostic criteria and genotyping was performed blind with respect to case–control status.

In conclusion, our findings suggest that Gpx1 Pro198Leu genotypes are significantly associated with the risk of diabetic peripheral neuropathy in patients with T2DM. These results require replication in future studies. The effect on observed genetic susceptibility is modest but might be important in the presence of other genetic factors. The study provides new clinically relevant information regarding genetic determinants of susceptibility to diabetic neuropathy.

## References

[CR1] American Diabetes Association (2013). Standards of medical care in diabetes. Diabetes Care.

[CR2] Brownlee M (2001). Biochemistry and molecular cell biology of diabetic complications. Nature.

[CR3] Brownlee M (2005). The pathology of diabetic complications: a unifying mechanism. Diabetes.

[CR4] Cao M, Mu X, Jiang C, Yang G, Chen H, Xue W (2014). Single- nucleotide polymorphisms of Gpx1 and MnSOD and susceptibility to bladder cancer: A systematic review and meta-analysis. Tumour Biology.

[CR5] Chen H, Yu M, Li M, Zhao R, Zhu Q, Zhou W (2012). Polymorphic variations in manganese superoxide dismutase (MnSOD), glutathione peroxidase-1 (GPX1), and catalase (CAT) contribute to elevated plasma triglyceride levels in Chinese patients with type 2 diabetes or diabetic cardiovascular disease. Molecular and Cellular Biochemistry.

[CR6] El Bghdady NA, Badr GA (2012). Evaluation of oxidative stress markers and vascular risk factors in patients with diabetic peripheral nephropathy. Cell Biochemistry and Function.

[CR7] Evans JL, Goldfine ID, Maddux BA, Grodsky GM (2002). Oxidative stress and stress-activated signaling pathways: A unifying hypothesis of type 2 diabetes. Endocrine Reviews.

[CR8] Faure P, Wiernsperger N, Polge C, Favier A, Halimi S (2008). Impairment of the antioxidant properties of serum albumin in patients with diabetes: Protective effects of metformin. Clinical Science (London).

[CR9] Flekac M, Skrha J, Hilgertova J, Lacinova Z, Jarolimkova M (2008). Gene polymorphisms of superoxide dismutase and catalase in diabetes mellitus. BMC Medical Genetics.

[CR10] Forsberg L, Faire U, Morgenstern R (2001). Oxidative stress, human genetic variation, and disease. Archives of Biochemistry and Biophysics.

[CR11] Hamanishi T, Furuta H, Kato H (2004). Functional variants in the glutathione peroxidase-1 (Gpx-1) gene are associated with increased intima-media thickness of carotid arteries and risk of macrovascular diseases in Japanese type 2 diabetic patients. Diabetes.

[CR12] Hansen RD, Krath BN, Frederiksen K, Tjonneland A, Overvad K, Roswall N (2009). GPX1 Pro(198)Leu polymorphism, erythrocyte GPx activity, interaction with alcohol consumption and smoking, and risk of colorectal cancer. Mutation Research.

[CR13] Houldsworth A, Hodgkinson A, Shaw S, Millward A, Demaine AG (2015). Polymorphic differences in the SOD-2 gene may affect the pathogenesis of nephropathy in patients with diabetes and diabetic complications. Gene.

[CR14] Hu YJ, Diamond AM (2003). Role of glutathione peroxidase 1 in breast cancer: Loss of heterozygosity and allelic differences in the response to selenium. Cancer Research.

[CR15] Jefferies S, Kote-Jarai Z, Goldgar D, Houlston R (2005). Association between polymorphisms of the Gpx1 gene and second primary tumours after index squamous cell cancer of the head and neck. Oral Oncology.

[CR16] Kasznicki J, Kosmalski M, Sliwinska A, Mrowicka M, Stanczyk M, Majsterek I (2012). Evaluation of oxidative stress markers in pathogenesis of diabetic neuropathy. Molecular Biology Reports.

[CR17] Kasznicki J, Sliwinska A, Kosmalski M, Merecz A, Majsterek I, Drzewoski J (2016). Genetic polymorphisms (Pro197Leu of Gpx1, +35 A/C of SOD1, -262 C/T of CAT), the level of antioxidant proteins (Gpx1, SOD1, CAT) and the risk of distal symmetric polyneuropathy in Polish patients with type 2 diabetes mellitus. Advances in Medical Sciences.

[CR18] Matsuno S, Sasaki H, Yamasaki H, Yamaoka H, Ogawa K, Nakatani M (2011). Pro198Leu missense polymorphism of the glutathione peroxidase 1 gene might be a common genetic predisposition of distal symmetric polyneuropathy and macrovascular disease in Japanese type 2 diabetic patients. Journal of Diabetes Investigation.

[CR19] Nemoto M, Nishimura R, Sasaki T, Hiki Y, Miyashita Y, Nishioka M (2007). Genetic association of glutathione peroxidase-1 with coronary calcification in type 2 diabetes: A case-control study with multi-slice computed tomography. Cardiovascular Diabetology.

[CR20] Oyenihi AB, Ayeleso AO, Mukwevho E, Masola B (2015). Antioxidant strategies in the management of diabetic neuropathy. BioMed Research International.

[CR21] Ramprasath T, Murugan PS, Kalaiarasan E, Gomathi P, Rathinavel A, Selvam GS (2012). Genetic association of glutathione peroxidase-1 (Gpx-1) and NAD(P)H: Quinine oxidoreductase 1 (NQO1) variants and their association of CAD in patients with type 2 diabetes. Molecular and Cellular Biochemistry.

[CR22] Ravn-Haren G, Olsen A, Tjonneland A, Dragsted LO, Nexo BA, Wallin H (2006). Associations between GPX1 Pro198Leu polymorphism, erythrocyte GPX activity, alcohol consumption and breast cancer risk in a prospective cohort study. Carcinogenesis.

[CR23] Robertson RP, Harmon J, Tran PO, Poitout V (2004). Beta-cell glucose toxicity, lipotoxicity, and chronic oxidative stress in type 2 diabetes. Diabetes.

[CR24] Rosenblat M, Aviram M (1998). Macrophage glutathione content and glutathione peroxidase activity are inversely related to cell-mediated oxidation of LDL: In vitro and in vivo studies. Free Radical Biology and Medicine.

[CR25] Said G, Lacroix C, Lozeron P, Ropert A, Plante V, Adams D (2003). Inflammatory vasculopathy in multifocal diabetic neuropathy. Brain.

[CR26] Suzen HS, Gucyener E, Sakalli O, Uckun Z (2010). CAT C-262T and GPX1 Pro198Leu polymorphisms in a Turkish population. Molecular Biology Reports.

[CR27] Tang TS, Prior SL, Li KW, Ireland HA, Bain SC, Hurel SJ (2012). Association between the rs1050450 glutathione peroxidase-1 (C > T) gene variant and peripheral neuropathy in two independent samples of subjects with diabetes mellitus. Nutrition, Metabolism and Cardiovascular Disease.

[CR28] Tang NP, Wang LS, Yang L, Gu HJ, Sun QM, Cong RH (2008). Genetic variant in glutathione peroxidase 1 gene is associated with an increased risk of coronary artery disease in Chinese population. Clinica Chimica Acta.

[CR29] Tesfaye S, Boulton AJ, Dyck PJ, Freeman R, Horowitz M, Kempler P, Toronto Diabetes Neuropathy Expert Group (2010). Diabetic neuropathies: update on definition, diagnostic criteria, estimation of severity, and treatments. Diabetes Care.

[CR30] Tsuzura S, Ikeda Y, Suehiro T, Ota K, Osaki F, Arii K (2004). Correlation of plasma oxidized low-density lipoprotein levels to vascular complications and human serum paraoxonase in patients with type 2 diabetes. Metabolism.

[CR31] Vats P, Sagar N, Singh TP, Banerjee M (2015). Association of superoxide dismutases (SOD1 and SOD2) and glutathione peroxidase 1 (Gpx1) gene polymorphisms with type 2 diabetes mellitus. Free Radical Research.

[CR32] Vincent AM, Russel JW, Low P, Feldman EL (2004). Oxidative stress in the pathogenesis of diabetic neuropathy. Endocrine Reviews.

[CR33] Vinik AI, Nevoret ML, Casellini C, Parson H (2013). Diabetic neuropathy. Endocrinology and Metabolism Clinics of North America.

[CR34] Vinik AI, Park TS, Stansberry KB, Pittenger GL (2000). Diabetic neuropathies. Diabetologia.

[CR35] Ybarra J, Pou JM, Romeo JH, Merce J, Jurado J (2010). Transforming growth factor beta 1 as a biomarker of diabetic peripheral neuropathy: Cross-sectional study. Journal of Diabetes and Its Complications.

